# Park exposure levels in older adults: the more significant influence of objective environment

**DOI:** 10.3389/fpsyg.2026.1945202

**Published:** 2026-10-21

**Authors:** Yashan Chen, Wanxin Zhuang, Ling Li, Guocheng Chen, Linsheng Huang

**Affiliations:** 1 College of Civil Engineering, Putian University, Putian, Fujian, China; 2 College of Environmental and Biological Engineering, Putian University, Putian, Fujian, China

**Keywords:** community park, physical activity, park facility, perceived environment, Xiamen, sedentary behavior, social interaction

## Abstract

**Background:**

Park exposure has been linked to multiple physical and mental benefits for older adults, and their actual exposure may be influenced by both objective and perceived environmental factors. However, it remains unclear which factors cause older adults’ perceived environment to diverge from the objective one, how such perceptions vary within this population, and, especially at the park scale, what the independent effects of these two types of environmental factors are and how much they relatively contribute.

**Objective:**

This study aimed to describe older adults’ perceptions of community park environments, compare the effects of objective versus perceived environmental factors on park exposure levels, and provide evidence for improving environmental perceptions and promoting park exposure among older adults.

**Methods:**

Taking 83 sites in Xiamen’s community parks as examples, this study measured 12 objective environmental factors and collected 404 questionnaires assessing older adults’ perceived environment and park exposure. Data were analyzed using Spearman’s correlation, nonparametric tests, and multiple linear regression.

**Results:**

Older adults perceive objective environmental factors in two distinct ways: a relatively accurate cognitive mapping and a biased cognitive translation. Blue view index and seating density are positive, effective factors that enhance perceived satisfaction, while sky view factor and travel distance are negative factors that lower satisfaction ratings. Perceived environmental factors did not show a significant predictive effect on the park exposure level among older adults. In contrast, objective environmental factors, including travel distance (*p* = 0.00), size of sub-area (*p* = 0.05), fitness facilities (*p* = 0.01), seating (*p* = 0.01), and amenity density (*p* = 0.04), were found to significantly influence the park exposure level.

**Conclusion:**

The findings of this study reveal key objective environmental factors associated with older adults’ perceived environment and park exposure. These findings can inform targeted and feasible interventions to improve older adults’ satisfaction with and engagement in community parks.

## 1 Introduction

Global population aging is accelerating, with the number of people aged 60 and older projected to double by 2050, posing serious challenges to public health and community services. Community parks are increasingly recognized as key spaces for supporting healthy aging (Moore et al., 2019), as they can promote physical activity and social interaction and thereby benefit older adults’ health (Gascon et al., 2015; Wedyan et al., 2024). However, these health benefits are not automatically realized: they depend on older adults’ actual exposure to park environments. Such exposure is shaped not only by objective environmental conditions but also by how older adults perceive their environment. Before examining what shapes such exposure, it is necessary to clarify how park exposure is conceptualized and measured. Addressing this issue is therefore essential for translating the recognized health benefits of community parks into real gains for older adults.

Park exposure refers to the degree and frequency of human interaction with a park (Jiang and Wang, 2025). Greater park exposure has been associated with higher levels of physical activity, improved mental health and psychological restoration, stronger social interaction and social cohesion, and better overall health outcomes among older adults (Rigolon et al., 2021; Wedyan et al., 2024; Wu et al., 2026). Importantly, mere park availability or accessibility does not equate to actual exposure, as older adults may not use an available park or may use it only briefly or in limited ways. Therefore, park exposure is typically measured by the frequency and duration of park visits, as well as the depth and breadth of participation in various park activities, in order to assess the “dose–response” relationship between parks and health (Kondo et al., 2018).

Park exposure is jointly shaped by factors across multiple levels, ranging from demographic characteristics to park features, community environment, and policy context (Lachowycz and Jones, 2013). Guided by this framework, older adults’ park exposure is conceptualized as an outcome of cross-level interactions among individual factors, objective environments, and perceived environments. Individual factors may shape older adults’ capacity to use parks (Lin and Feng, 2023), while objective and perceived environments may influence their motivation to use parks (Li et al., 2026a). These three sets of factors jointly shape whether and how older adults are actually exposed to parks. The objective environment of community parks refers to the set of spatial elements and facility characteristics within park boundaries that are directly observable or physically measurable. Perceived environment represents an individual’s subjective understanding and psychological experience of the objective environment (Peters et al., 2020), and is shaped by age, social background, and other individual characteristics and may deviate from objective environmental conditions (Ma and Dill, 2017; Tendron et al., 2026). Because such perceptual differences are particularly pronounced in later life (Li et al., 2026b), older adults’ perceptions of park environments cannot be assumed to align with objective conditions, making it necessary to pay particular attention to their needs and experiences.

Older adults are in a unique stage of life, and their sensory, mobility, and safety sensitivities may lead them to perceive the objective environment inaccurately. Due to the general decline in sensory functions, their ability and quality of obtaining information from the environment differ from those of other populations (Shayman et al., 2024). Older adults are also more sensitive to obstacles and safety issues in the environment (Kanav and Kumar, 2024). Places with excessively complex environments, such as those with a higher number of intersections and turns (Manley et al., 2021; Mohamed et al., 2022), may increase the perceived distance and reduce older adults’ sense of support and satisfaction (Cassarino and Setti, 2016). In addition, older adults’ sensory experiences are closely linked to their daily activities and overall satisfaction (Trivic, 2021). However, although existing studies have documented discrepancies between objective and perceived environments, it remains unclear which specific environmental factors are most likely to lead to perceptual inaccuracy among older adults. Meanwhile, existing research has largely focused on differences between age groups but has paid insufficient attention to perceptual differences within the older population, making it difficult to explain why older adults perceive the same objective environment differently.

Both objective and perceived environmental factors have been found to be associated with older adults’ park use. Objective environmental factors, such as accessibility (Kanav and Kumar, 2024), facility provision (Rung et al., 2011; Cohen et al., 2012), park area (Levy-Storms et al., 2018), greenery and naturalness (Taheri et al., 2026), and shading (Hami et al., 2025), have been linked to older adults’ park exposure, including whether they use parks, how frequently they visit, and how long they stay. Perceived environmental factors, including perceived safety (Cornu et al., 2024), accessibility (Wang et al., 2025), facility appropriateness (Van Puyvelde et al., 2023), natural esthetics (Wu et al., 2024), environmental quality, and social environment (Wang et al., 2025), have likewise been shown to shape older adults’ park exposure and their persistence in park activities. Some studies suggest that objective environmental factors have stronger associations, as objective measures better reflect actual environmental conditions (Lin and Moudon, 2010), whereas others point out that subjective factors have a more significant influence (Kim, 2016; Ayalon, 2023); under similar environmental conditions, higher perceived environmental evaluations are more likely to predict actual use behavior (Poturalska et al., 2026). Such inconsistency may arise from differences in measurement indicators, research methods, exposure types, and environmental types (Peters, M.). To date, studies that simultaneously examine and compare objective and perceived environments remain relatively limited, and no consistent conclusions have yet been reached. Furthermore, most studies have focused on the neighborhood or community level, while at the park scale, the independent effects of these two environmental factors on different dimensions of park exposure and their relative contributions remain unclear.

To address the explanatory limitations of existing research on the primary factors influencing older adults’ park exposure levels, this study takes 83 sub-areas from eight community parks in Xiamen as examples. Starting from objective measurements of the sub-area environment, older adults’ perceptual evaluations, and their park exposure levels, the study explores older adults’ perceptual characteristics of the park environment and the main factors influencing park exposure levels, thereby providing refined evidence for age-friendly park design and enhancing the health benefits of parks. Based on the interactive perspective of “objective environment” and “subjective perception,” this study aims to answer the following questions:

(1) Which specific environmental factors are most likely to cause perceptual inaccuracy among older adults?
(2) How do individual differences lead older adults to perceive the park environment differently?
(3) Which has a greater relative contribution to park exposure levels: the objective park environment or older adults’ perceptions?

## 2 Materials and methods

### 2.1 Study area

Xiamen, located on the southeastern coast of China, has long been committed to the development of high-quality park green spaces and can provide ample and diverse samples of community parks. The study was conducted in Siming District and Huli District, the two areas of Xiamen with the highest density of older adults. Through a preliminary survey, eight community parks with high visitation rates among older adults were selected from these two districts for on-site investigation and questionnaire surveys (Figure 1).

**Figure 1:**
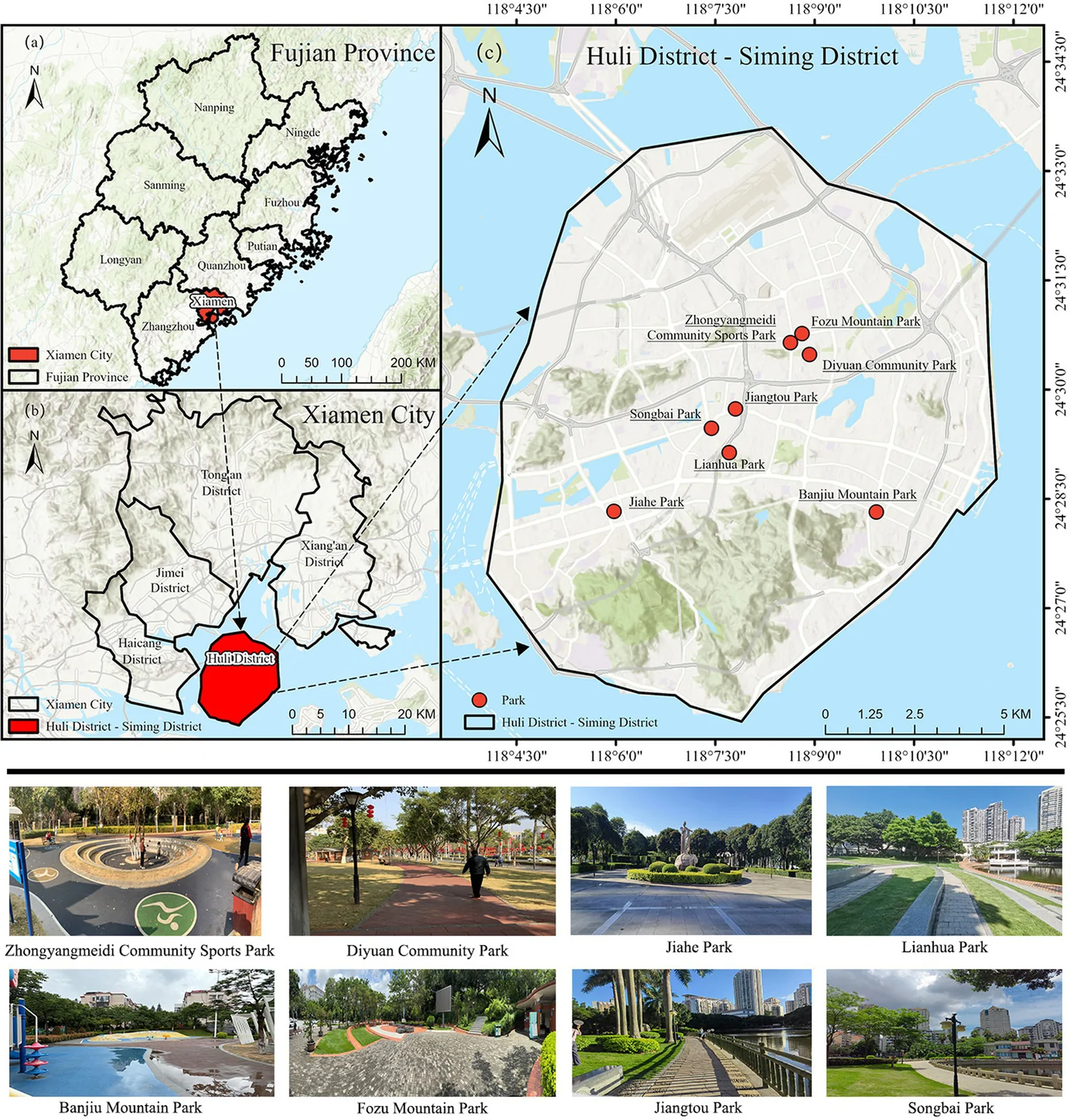
Distribution and current status of parks in the study area.

As the basic spatial units of parks, sub-areas differ in spatial layout, landscape design, and facility provision, which directly affect older adults’ frequency of use and environmental perception. This study takes individual sub-areas within a park, rather than the park as a whole, as the basic unit of analysis, aiming to avoid homogenizing park environments that are inherently spatially heterogeneous. This analytical scale has been applied in similar environment–behavior studies (Sant’Anna et al., 2024; Hu et al., 2026). The sub-areas were selected according to the following criteria: (1) each sub-area had to be a site equipped with fixed facilities such as seating and fitness equipment, thereby possessing the physical conditions to attract lingering, or an activity space where older adults were actually observed during the observation period (Chen et al., 2026b); (2) paths were excluded from the sampling frame because their environmental factor composition is fundamentally different from that of lingering-oriented sites, and they were not the core focus of this study; and (3) each sub-area had to be internally consistent in its physical attributes and spatial structure. Boundaries were delineated based on conspicuous features such as pavement edges or building outlines. In the absence of such features, the boundaries were defined by observed areas of human activity. All the above criteria were implemented based on objectively measured on-site conditions, thereby minimizing subjective intervention by the researchers. Accordingly, a total of 83 sub-areas were ultimately identified (Table 1; Figure 2). The sub-areas varied considerably in size (range: 32.0–1118.4 m^2^; mean = 327.9, SD = 330.9). This variation reflects the heterogeneity of different functional spaces within the park in terms of actual layout, but it also suggests that scale effects need to be taken into account in subsequent analyses.

**Table 1:** Overview of community parks and number of sub-areas.

District	Park name	Construction year	Park area/hm^2^	Number of sub-areas
Siming district	Jiahe Park	1988	1.6	3
	Lianhua Park	1988	2.2	11
	Banjiu Mountain Park	2019	1.4	8
	Songbai Park	1996	7.2	20
Huli district	Diyuan Community Park	2016	1.5	8
	Zhongyangmeidi Community Sports Park	2018	1.3	6
	Jiangtou Park	2003	6.0	18
	Fozu Mountain Park	2021	1.0	9
Total			28.6	83

**Figure 2:**
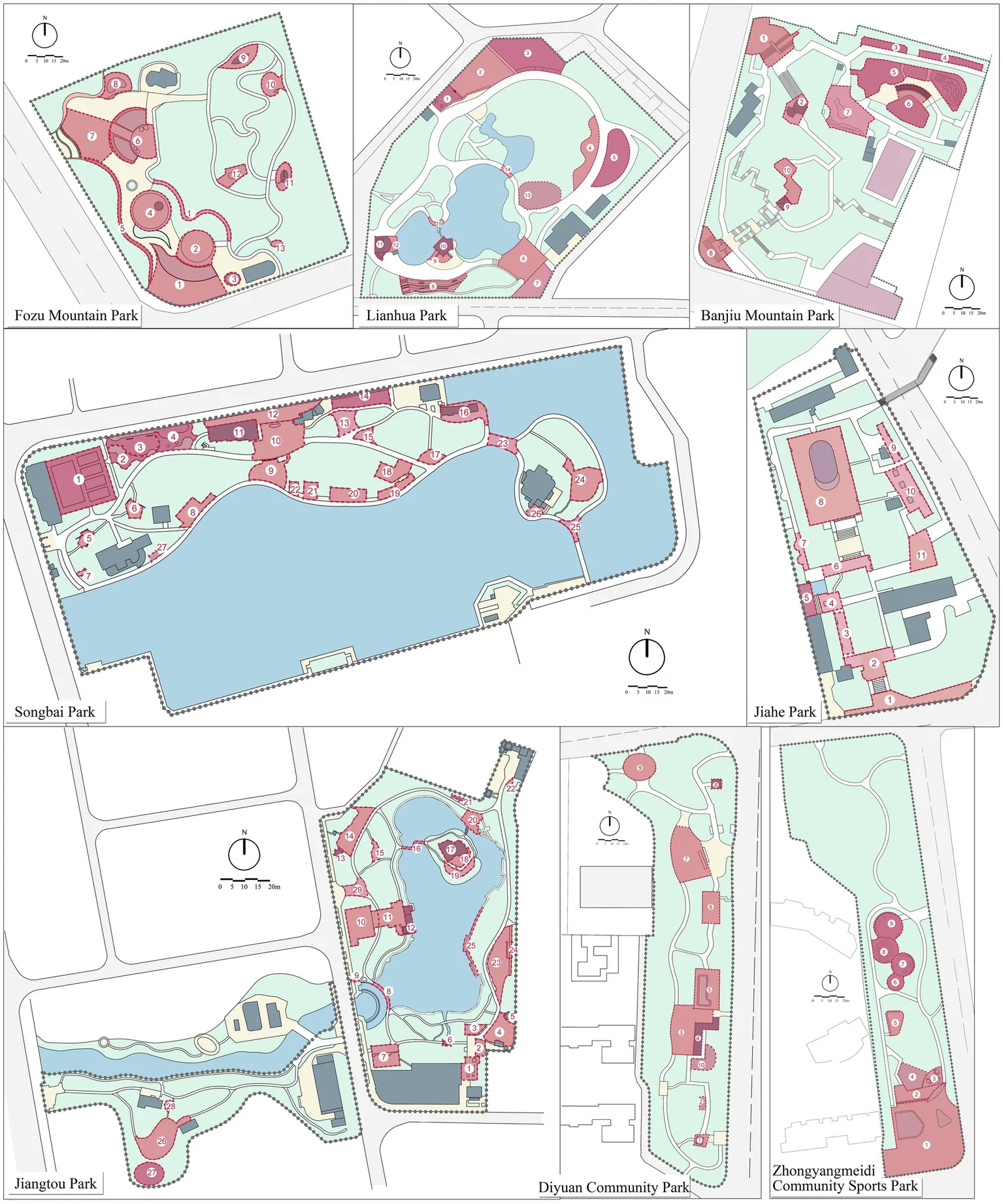
Sub-areas of the eight parks.

Data collection was conducted from July to August 2025. At random times throughout the day (morning and afternoon), older adults (aged 60 and over) were approached at randomly selected times during morning and afternoon periods at the target sub-areas and invited to complete the questionnaire, with at least 4 complete questionnaires collected per sub-area. To ensure data coverage, efforts were made to invite users engaging in different activities to complete the survey, avoiding data concentration within a single activity group. Because some older adults may be illiterate, suffer from vision loss, or have difficulty understanding questionnaire items, interviewers were required to assist them in answering the questions during the process. A total of 404 valid questionnaires were collected from 83 sub-areas. The research adhered to relevant ethical guidelines, ensuring participants’ informed consent and privacy protection.

### 2.2 Variable selection and questionnaire design

#### 2.2.1 Objective environmental factors

Bedimo-Rung et al. (2005) proposed a conceptual model of parks and physical activity that categorizes park environmental characteristics into six dimensions: features, condition, access, esthetics, safety, and policies. This model has been applied in studies of park exposure among older adults. Guided by this model and taking into account the unique sensory, mobility, and safety sensitivities of older adults, the study selected 11 objective environmental factors that are most relevant to older adults’ park exposure from these six dimensions. For the access dimension, travel distance was selected as an objective factor of accessibility. For the esthetics dimension, green view index, blue view index, and sky view factor were selected to reflect older adults’ objective exposure to visual quality. For the features dimension, seating density, amenity density, and fitness facility density were selected, covering three types of behavioral support: resting, convenience, and activity. For the condition dimension, sound level and area were selected to reflect acoustic environmental quality and spatial sufficiency, respectively. For the safety dimension, slope and accessible facilities were selected to reflect circulation safety and accessibility safety, respectively. It should be noted that the policy dimension was not included in this study because it is difficult to capture through objective measurement (Table 2).

**Table 2:** Quantification of objective environmental factors.

Factor	Code	Measurement (unit)	Data source/Tool
Travel distance	DI	The actual distance between the sub-area and the respondent’s place of residence (m)	GIS
Sound level	NO	Measured equivalent continuous A-weighted sound level at the site (dB)	Sound level meter
Slope	SL	Mean slope of pathways leading to the sub-area (°)	Inclinometer
Area	AR	The size enclosed by the sub-area boundaries (m^2^)	Master plan
Green view index	GVI	Ratio of green pixels to total pixels (−)	On-site photos
Blue view index	BVI	Ratio of water pixels to total pixels (−)	
Sky view factor	SVF	Ratio of sky pixels to total pixels (−)	
Seating density	SF	Total number of seats divided by the size of the sub-area. The number of seats in standard seating is counted at 100%, while the number of seats in auxiliary seating is counted at 50% (units/m^2^)	Field observations
Amenity density	AM	The number of amenities (trash cans, water dispensers, coat racks, etc.) divided by the size of the sub-area (units/m^2^)	
Fitness facility density	RF	The number of fitness facilities (such as exercise equipment and children’s slides) divided by the size of the sub-area (units/m^2^)	
Accessible facilities	AC	Number of accessible facility types (types)	

Among these factors, travel distance and sound level vary depending on the respondent, the time, and the location; therefore, they are measured concurrently with the questionnaire. Travel distance is calculated by including a relevant question in the survey, obtaining the respondent’s location, and then using GIS to measure the actual distance between the two points. Sound level was measured using an AWA5688 sound level meter, with the microphone placed at a height of 1.2 m. The measurement point was located at the geometric center of the sub-area where the respondents were staying. Each measurement lasted 3–5 min, and the instantaneous A-weighted sound level was recorded at 1-s intervals. The equivalent continuous A-weighted sound level over the measurement period was then calculated through time integration.

In addition, green view index and blue view index were measured by taking photographs from the center of the sub-area at human eye-level perspective, looking in four directions: 0°, 90°, 180°, and 270°. A semantic segmentation software based on a deep learning fully convolutional network trained on the ADE_20K dataset was used to identify landscape elements in photographs and calculate the percentage of environmental attributes within each photograph. The green view index quantifies the percentage of pixels occupied by trees, shrubs, grass, and flowers, while the blue view index measures the percentage of pixels occupied by water areas (rivers, lakes, oceans, and artificial water features). The value for each sub-area was based on the average of four photos. Sky view factor was measured by placing a camera with a fixed-focus fisheye lens horizontally on the ground at the central sampling point of the sub-area, with the lens pointing upward toward the sky. The pixel ratio of the sky was then calculated using the same semantic segmentation software.

#### 2.2.2 Perceived environmental factors

The perceived environmental factors in this study were derived from the Age-Friendly Community Parks Survey (AF-CPS), a measurement tool developed specifically for assessing older adults’ perceptions of community park environments (Xiang and Mei, 2024). The AF-CPS categorizes older adults’ psychological perceptions of the park environment into five domains: (1) perceptions of the park’s sounds, lighting, and site features; (2) perceptions of environmental safety; (3) perceptions of accessibility, enjoyment, esthetics, and attractiveness; (4) perceptions of management; and (5) social and community support (Xiang and Mei, 2024). To ensure dimensional consistency between subjective perceptual factors and objective environmental factors, this study included only those perceptual factors that could be matched with objective environmental factors, and excluded factors that pertained to the entire park rather than the sub-area scale. A total of 12 perceived environmental attributes were ultimately extracted, including micro-climatic comfort, acoustic comfort, esthetics, psychological safety, physical safety, site convenience, seating convenience, fitness facility convenience, amenity convenience, family support, community support, and policy support.

While the AF-CPS provides a validated operational basis for factor selection, its theoretical grounding in classic environmental psychology requires further clarification. Drawing on Attention Restoration Theory (ART) and Stress Recovery Theory (SRT), micro-climatic comfort and acoustic comfort can reduce environmental stress and enhance immediate comfort during park use (Wu et al., 2025), thereby increasing older adults’ willingness to stay. Esthetics serves as a core trigger of “fascination” in ART, strengthening environmental preference and park choice (Fischer et al., 2018). Psychological safety and physical safety, meanwhile, constitute preconditions for restorative experience and park use, as unsafe environments discourage people from visiting parks (Pérez-Tejera et al., 2022). These attributes primarily reflect whether the park environment allows older adults to feel at ease, restore attention, and relieve stress. In the attachment pathway, which draws on environmental preference and place attachment theory, perceived convenience of site, seating, fitness, and leisure facilities reflects functional place dependence, thereby promoting repeated use and longer stays (Chen et al., 2022); family support and community support correspond to affective place dependence, helping to sustain continued use (Chen et al., 2022); and policy support is an important antecedent of place attachment, helping to stabilize expectations of park use.

#### 2.2.3 Park exposure level

According to existing research, older adults’ park usage involves two dimensions that may influence health: exposure intensity and interaction intensity (Kondo et al., 2018; Jiang and Wang, 2025). Exposure intensity includes visit frequency and visit duration (Table 3), while interaction intensity can be divided into activity intensity and social activeness (Yessoufou et al., 2020).

**Table 3:** Park exposure indicators, items, and scoring methods.

Dimension	Indicator	Item	Options and scoring
Exposure intensity	Visit frequency	How often do you usually come to this sub-area?	Less than once a week = 1; 1–3 times a week = 2; 4–6 times a week = 3; Once a day or more = 4
	Visit duration	How long do you usually stay at this sub-area?	Within 10 min = 1; 11–30 min = 2; 31–60 min = 3; More than 60 min = 4
Interaction intensity	Activity intensity	What are your main activities in this sub-area?	Sedentary behavior = 1; Light-intensity physical activity = 2; Moderate-intensity physical activity = 3; Vigorous-intensity physical activity = 4
	Social activeness	How active are you socially in this sub-area?	Low social activeness = 1; Moderate social activeness = 2; High social activeness = 3

To comprehensively characterize older adults’ park exposure levels, this study constructed two continuous indicators based on park visit frequency, duration per visit, activity intensity, and social activeness. Existing physical activity research typically regards frequency, duration, and intensity as the three core dimensions for measuring overall activity levels, and commonly uses the product of the first three as the quantitative score for overall activity level (Wu et al., 2026). Research on social interaction, in turn, indicates that the level of social interaction in parks is related not only to whether interaction occurs, but also to interaction frequency, duration, and degree of interaction (Chen et al., 2026a). Therefore, within the same park exposure framework, this study defines the composite physical activity index and the composite social activeness index as follows:


$$\mathrm{PA}I_{i}=F_{i}\times D_{i}\times I_{i}$$

$$\mathrm{SA}I_{i}=F_{i}\times D_{i}\times S_{i}$$

Where *PAI*_i_ and *SAI*_i_ represent the composite physical activity index and the composite social activeness index of the i-th respondent, respectively; F_i_ is the assigned value for visit frequency, D_i_ is the assigned value for visit duration, I_i_ is the assigned value for activity intensity, and S_i_ is the assigned value for social activeness.

#### 2.2.4 Survey design and data collection

The questionnaire consists of three sections: respondents’ demographic characteristics, level of park exposure, and perceived environment evaluation. Demographic characteristics include three categories: gender, age, and educational level. Park exposure encompasses four items: visit frequency, visit duration, activity intensity, and social activeness. The perceived environment evaluation consists of a total of 12 items, all of which are phrased in positive terms. A 5-point Likert scale is used for measurement, with scores ranging from 1 (strongly disagree) to 5 (strongly agree).

The reliability and validity tests of the pilot survey (n = 105) showed that the scale had a Cronbach’s α of 0.77, a KMO value of 0.80, and a Bartlett’s test of sphericity *p* < 0.001, meeting the prerequisites for factor analysis. Through unrotated principal component analysis, three factors with eigenvalues greater than 1 were extracted. Among them, the factor loading of “family support” was below 0.4, while “microclimatic comfort,” “esthetics,” and “site convenience” exhibited cross-loadings on multiple factors (all greater than 0.4). None of these four factors met the independence requirement for factor attribution, and they were therefore removed. The remaining eight factors were retained for subsequent analysis: acoustic comfort, psychological safety, physical safety, seating convenience, fitness facility convenience, amenity convenience, community support, and policy support.

The second analysis used rotated principal component analysis and extracted two factors with eigenvalues greater than 1, which together explained 55.88% of the total variance. The first factor covered six factors—acoustic comfort, psychological safety, physical safety, seating convenience, fitness facility convenience, and amenity convenience—reflecting older adults’ overall evaluation of the appeal of environmental functionality and safety, and was named “perceived attractiveness.” The second factor covered two factors, community support and policy support, reflecting older adults’ perception of social connectedness, and was named “perceived support” (Table 4). It should be noted that the removal of the “esthetics” item does not represent a theoretical rejection. Although it holds an important position in restorative environment theory (Kaplan and Kaplan, 1989), in the older adult sample of this study, esthetic evaluation was highly intertwined with functional judgment (cross-loading > 0.4), making it difficult to exist as an independent dimension; its theoretical effect has been indirectly reflected through the composite construct of the “perceived attractiveness” factor.

**Table 4:** Perceived environment evaluation scale.

Factor	Code	Variable	Item
Perceived attractiveness	PA1	Acoustic comfort	The soundscape here makes me feel comfortable.
	PA2	Psychological safety	There are no external dangers here, so I feel very safe.
	PA3	Physical safety	There are anti-slip and fall prevention measures here, which makes me feel very safe.
	PA4	Seating convenience	The seating here is very convenient for me.
	PA5	Fitness facility convenience	The fitness facilities and slides here are very convenient for me.
	PA6	Amenity convenience	The amenities here are very convenient for me.
Perceived support	PS1	Community support	The community environment and sense of belonging support my decision to be here.
	PS2	Policy support	Government policies have supported my decision to come here.

### 2.3 Data analysis

First, descriptive statistical analyses were conducted on older adults’ personal characteristics, park exposure characteristics, and perceived environmental evaluations. Second, Spearman correlation analysis was used to examine the relationships between objective environmental factors and perceived environmental factors. Third, the Mann–Whitney U test (for binary variables such as gender) and the Kruskal–Wallis test (for multi-category variables such as age group) were used to compare differences in perceived environment across different groups. Both methods are non-parametric tests whose principles are based on ranks rather than raw values; therefore, they do not rely on the assumption of normal distribution and are suitable for the skewed perceived evaluation data in this study. If the test results were statistically significant (*p* < 0.05), Wilcoxon rank-sum tests were further employed for *post hoc* pairwise comparisons to identify differences among groups.

Fourth, to investigate the relative contributions of objective environmental factors and perceived environmental factors to older adults’ park exposure levels, this study constructed four multiple linear regression models. Models 1 and 2 used the composite physical activity index as the dependent variable, while Models 3 and 4 used the composite social activeness index as the dependent variable. Models 1 and 3 included objective environmental factors as independent variables, whereas Models 2 and 4 included perceived environmental factors as independent variables. All models included individual characteristics (gender, age, and education) as control variables to rule out the potential confounding effects of demographic characteristics. The differentiated design of the four models was intended to separately estimate the independent explanatory power of the two categories of factors—objective environment and perceived environment —for the same dependent variable, thereby providing a statistical basis for subsequently comparing the relative importance of the two categories of factors. Because the two dependent variables (composite physical activity index and composite social activeness index) both exhibited substantial positive skew (skewness > 1), natural log transformations were applied before analysis to ensure residual normality. Multicollinearity diagnostics were conducted for all regression models (VIF < 5), indicating that there was no severe collinearity problem among the independent variables.

## 3 Results

### 3.1 Descriptive statistics

Of the 404 respondents (Table 5), 49.5% were male, and 50.5% were female, representing a balanced distribution. Most of the population is aged 60–69, accounting for 51.2% of the total. The majority of participants (88.9%) had an educational level of secondary school or lower. In terms of park exposure, most older adults visit parks at least once a day (61.4%), and the duration of each visit is typically more than 1 h (55%). Overall, the exposure exhibits characteristics of high frequency and long duration. The total amount of physical activity (52.5%) was slightly higher than sedentary behavior (47.5%), with physical activity primarily consisting of moderate-intensity activities. Regarding social activeness, the majority (54.7%) reported high levels, while moderate ones were less common (33.2%).

**Table 5:** General characteristics of older adult respondents in community parks.

General characteristics	Variable	Number	Percentage (%)
Gender	Male	200	49.5
	Female	204	50.5
Age	60–69	207	51.2
	70–79	161	39.9
	≥80	36	8.9
Education level	Primary school or below	161	39.9
	Secondary school	198	49.0
	Junior college, bachelor’s degree, or above	45	11.1
Visit frequency	Less than once a week	8	2.0
	1–3 times a week	67	16.6
	4–6 times a week	81	20.0
	Once a day or more	248	61.4
Visit duration	Within 10 min	1	0.2
	11–30 min	49	12.1
	31–60 min	132	32.7
	More than 60 min	222	55.0
Activity intensity	Sedentary behavior	192	47.5
	Light-intensity physical activity	57	14.1
	Moderate-intensity physical activity	151	37.4
	Vigorous-intensity physical activity	4	1.0
Social activeness	Low	134	33.2
	Moderate	49	12.1
	High	221	54.7

In the perceived environment evaluation (Figure 3), community support received the highest mean score (X^−^ = 4.48), followed by policy support (X^−^ = 4.37) and psychological safety (X^−^ = 4.35). The average scores for fitness facility convenience (X^−^ = 3.82), seating convenience (X^−^ = 3.86), and amenity convenience (X^−^ = 4.02) were the lowest, while these three items also had the highest standard deviations.

**Figure 3:**
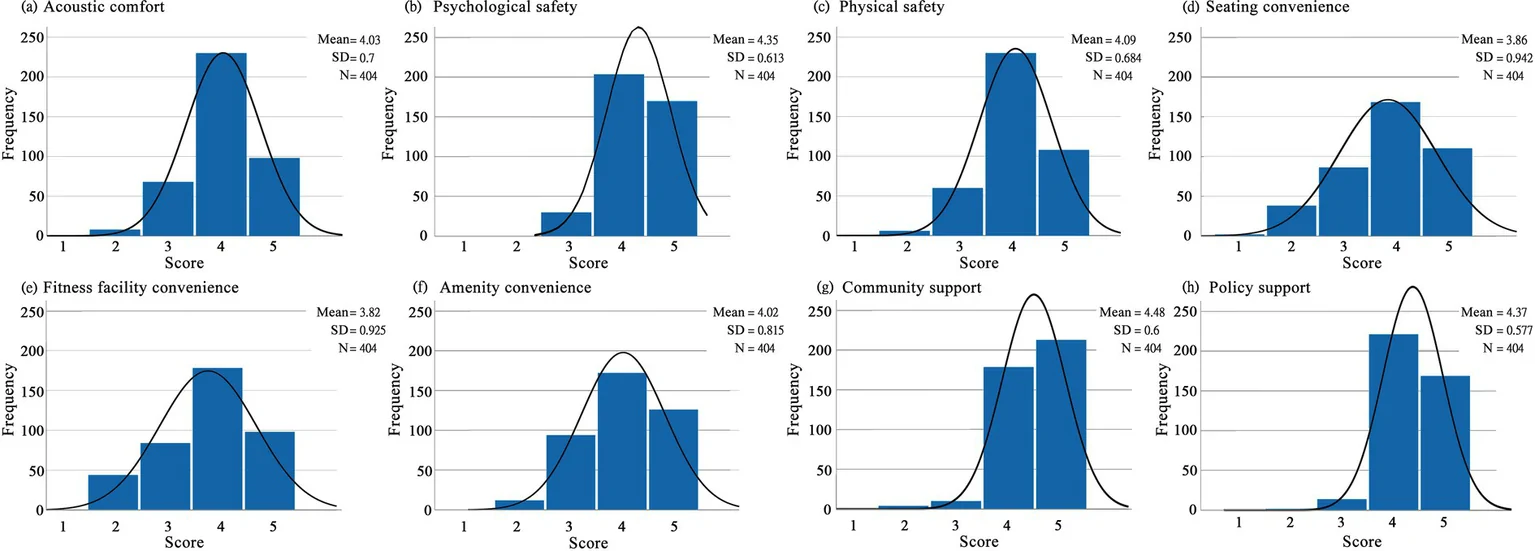
Scores for perceived environment evaluation.

### 3.2 Correlation analysis

Each perceived environmental factor is associated with specific objective environmental factors (Figure 4). Seating convenience was significantly positively correlated with various objective environmental factors, including seating density (*β* = 0.23, *p* < 0.01), blue view index (*β* = 0.20, *p* < 0.01), and amenity density (*β* = 0.16, *p* < 0.05), and was significantly negatively correlated with sky view factor (*β* = −0.14, *p* < 0.05). The factors associated with amenity convenience were the same as those associated with seating convenience. Acoustic comfort was found to be significantly positively correlated with blue view index (*β* = 0.27, *p* < 0.01) and amenity density (*β* = 0.15, *p* < 0.05). Psychological safety was positively correlated only with blue view index (*β* = 0.14, *p* < 0.05). Physical safety was positively correlated with seating density (*β* = 0.29, *p* < 0.01) and blue view index (*β* = 0.23, *p* < 0.01), and was significantly negatively correlated with sky view factor (*β* = −0.20, *p* < 0.01). Fitness facility convenience was positively correlated only with seating density (*β* = 0.16, *p* < 0.05) and negatively correlated with sky view factor (*β* = −0.16, *p* < 0.05), but it did not show a significant association with fitness facility density. Community support was positively correlated with seating density (*β* = 0.17, *p* < 0.05) and negatively correlated with slope (*β* = −0.22, *p* < 0.01) and travel distance (*β* = −0.20, *p* < 0.01). Policy support was significantly positively correlated with seating density (*β* = 0.22, *p* < 0.01) and sky view factor (*β* = 0.21, *p* < 0.01), and negatively correlated with sub-area size (*β* = −0.15, *p* < 0.05). Overall, blue view index and seating density were positive factors most closely associated with the greatest number of perceived environmental factors, whereas sky view factor was the negative factor most strongly associated with the greatest number of perceived environment factors. Green view index, accessible facilities, and fitness facility density showed no significant correlation with perceived environmental factors. In addition, Figure 4 showed that there was no significant correlation between sub-area size and travel distance (*β* = 0.07, *p* > 0.05), indicating that larger sub-areas did not correspond to shorter or longer travel distances. This result preliminarily suggests that differences in sub-area scale did not introduce systematic bias into distance measurement.

**Figure 4:**
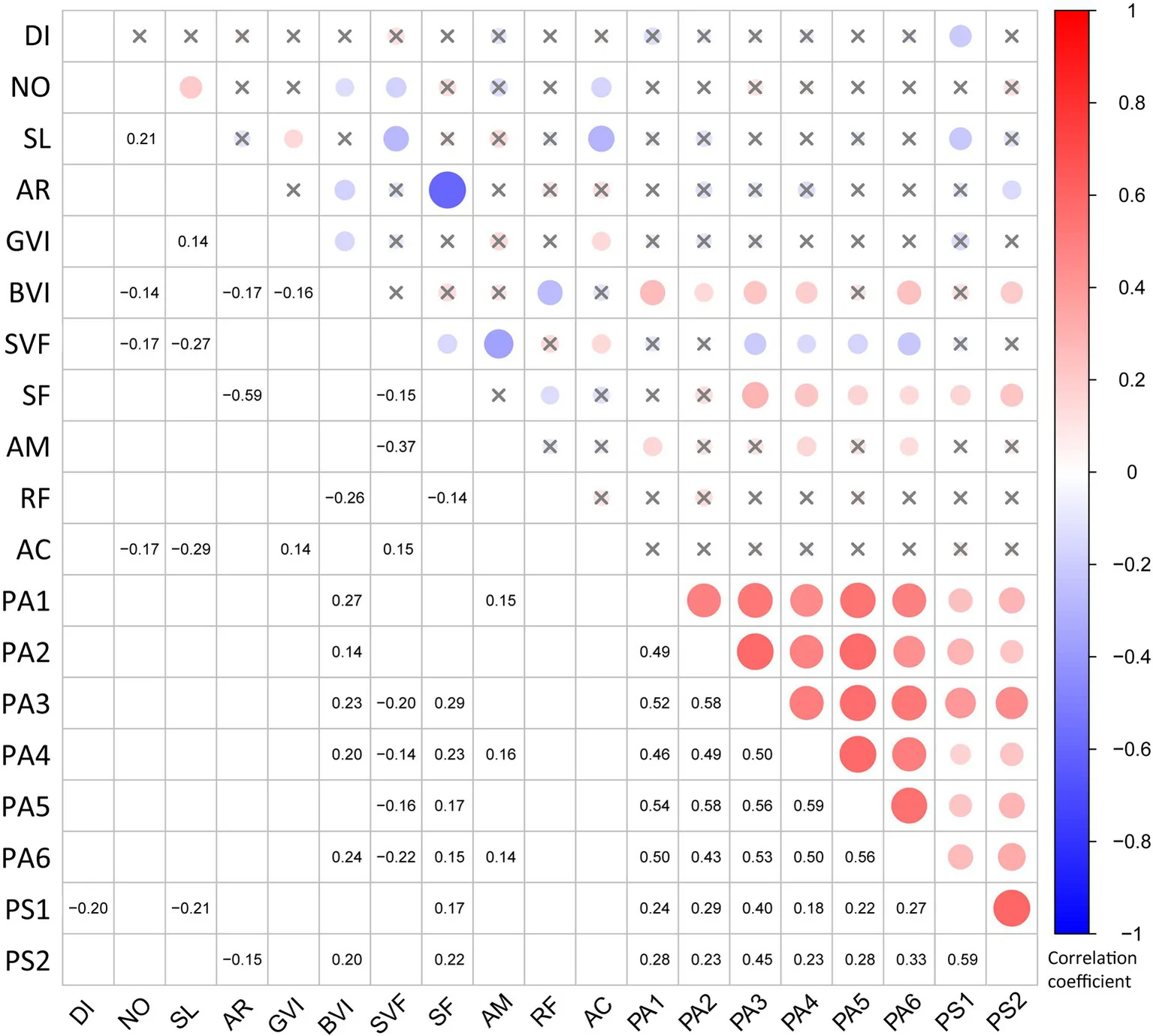
The correlation between objective environmental factors and perceived environmental factors [Circle color indicates correlation type (red: positive; blue: negative)]. Circle diameter is inversely proportional to the *p*-value (larger diameter = smaller *p*-value).

### 3.3 Nonparametric tests

#### 3.3.1 Different demographic characteristics

There are gender differences in older adults’ perceptions of acoustic comfort, psychological safety, and seating convenience (Table 6). Women rated all three items higher than men. Significant differences were observed only in the perception of psychological safety across different age groups, with older adults aged 80 and above reporting higher ratings than those aged 70–79 (Figure 5a). Differences in educational level were associated with significant differences in perceptions of fitness facility convenience, amenity convenience, and community support. Based on the results of post-hoc multiple comparisons, higher levels of education were associated with lower ratings of the fitness facility convenience (Table 7; Figure 5c). Respondents with a junior college, bachelor’s degree, or above also rated amenity convenience and community support significantly lower than the other two groups (Figures 5d,e). Although there is a clear trend of differences in acoustic environments and policy support across different educational levels, the specific differences between groups did not reach statistical significance (Figures 5b,f).

**Table 6:** Testing for gender differences in perceived environmental factors.

Statistic	PA1	PA2	PA3	PA4	PA5	PA6	PS1	PS2
Mann–Whitney U	17,738	17,664	20,198	18,000	18,900	18,606	19,146	20,172
Wilcoxon W	37,838	37,764	40,298	38,100	39,000	38,706	39,246	40,272
Z	−2.542	−2.613	−0.193	−2.159	−1.355	−1.63	−1.218	−0.222
Asymptotic Sig. (2-tailed)	0.011^*^	0.009^**^	0.847	0.031^*^	0.175	0.103	0.223	0.824

*p < 0.05; **p < 0.01.

**Figure 5:**
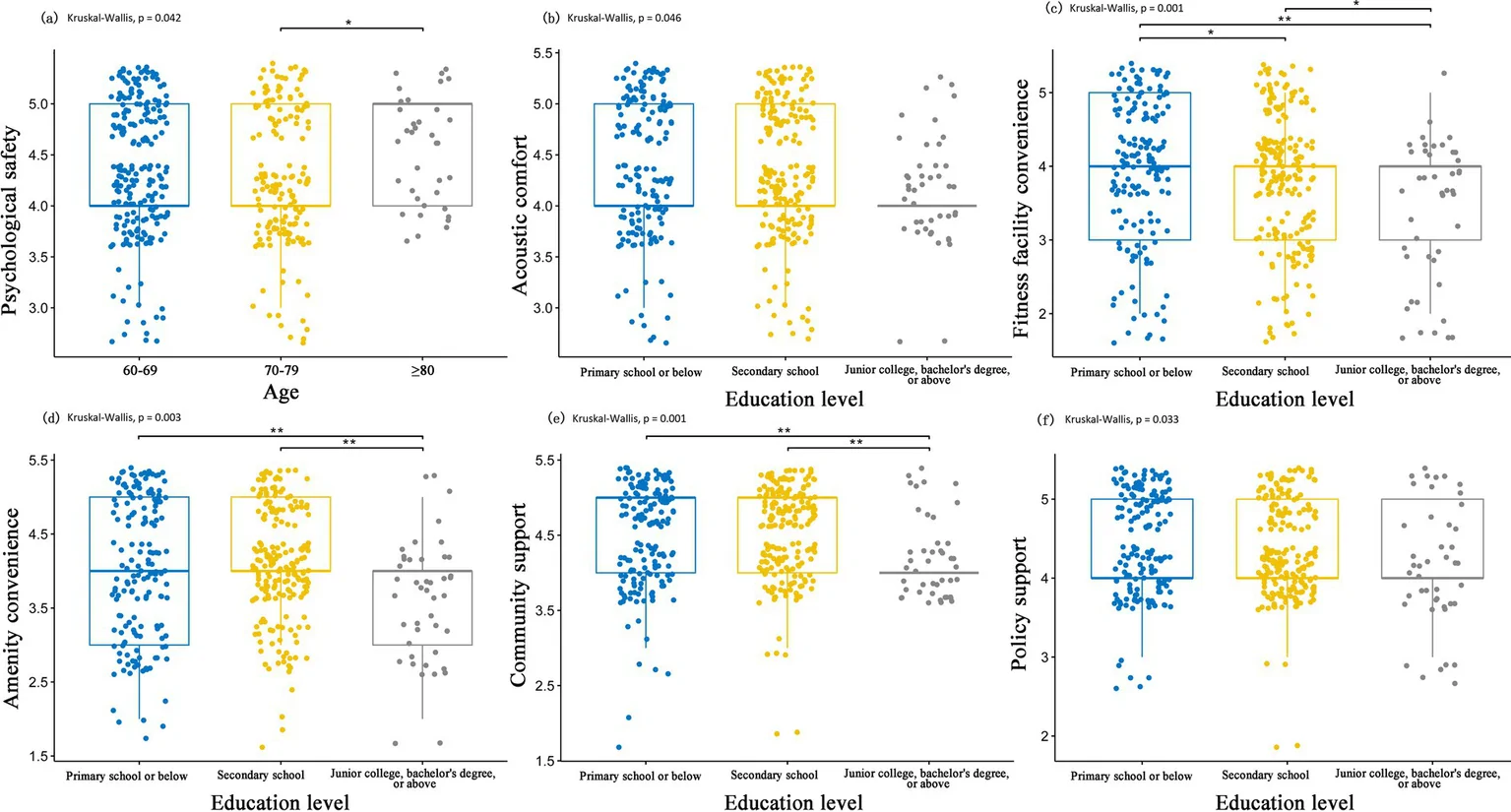
Post-hoc multiple comparisons across different demographic groups (*p < 0.05, **p < 0.01). **(a)** Differences in psychological safety among age groups. **(b)** Differences in acoustic comfort across education levels. **(c)** Differences in fitness facility convenience across education levels. **(d)** Differences in amenity convenience across education levels. **(e)** Differences in community support across education levels. **(f)** Differences in policy support across education levels.

**Table 7:** Testing for differences in perceived environmental factors across different ages and education levels.

Statistic	PA1	PA2	PA3	PA4	PA5	PA6	PS1	PS2
Grouping variable: age								
Kruskal-Wallis H	3.729	6.356	1.496	1.967	0.702	0.785	0.203	0.232
Asymptotic Sig.	0.155	0.042^*^	0.473	0.374	0.704	0.675	0.904	0.890
Grouping variable: education level								
Kruskal-Wallis H	6.174	5.59	3.333	3.176	13.221	11.834	14.628	6.796
Asymptotic Sig.	0.046^*^	0.061	0.189	0.204	0.001^**^	0.003^**^	0.001^**^	0.033^*^

*p < 0.05; **p < 0.01.

#### 3.3.2 Different parks exposure

Differences in perceived environment evaluations were observed across varying levels of visit frequency, activity intensity, and social activeness (Table 8); however, such differences were not found among groups with different visit durations. There are significant differences in seating convenience based on visit frequency. According to the results of post-hoc multiple comparisons (Figure 6a), older adults who visit the park less than once a week rated seating convenience significantly higher than the other three frequency groups, while those who visit 1–3 times a week rated seating convenience significantly lower than those who visit once a day or more.

**Table 8:** Testing for differences in perceived environmental factors across different park exposure.

Statistic	PA1	PA2	PA3	PA4	PA5	PA6	PS1	PS2
Grouping variable: visit frequency								
Kruskal-Wallis H	4.751	1.471	3.161	15.382	6.111	4.624	2.685	5.367
Asymptotic Sig.	0.191	0.689	0.367	0.002^**^	0.106	0.201	0.443	0.147
Grouping variable: activity intensity								
Kruskal-Wallis H	3.925	18.78	20.174	7.794	10.774	22.98	0.479	1.733
Asymptotic Sig.	0.270	<0.001^**^	<0.001^**^	0.050	0.013^*^	<0.001^**^	0.923	0.630
Grouping variable: social activeness								
Kruskal-Wallis H	11.135	5.54	4.951	1.539	4.515	2.68	6.397	28.187
Asymptotic Sig.	0.004^**^	0.063	0.084	0.463	0.105	0.262	0.041^*^	<0.001^**^

*p < 0.05; **p < 0.01.

**Figure 6:**
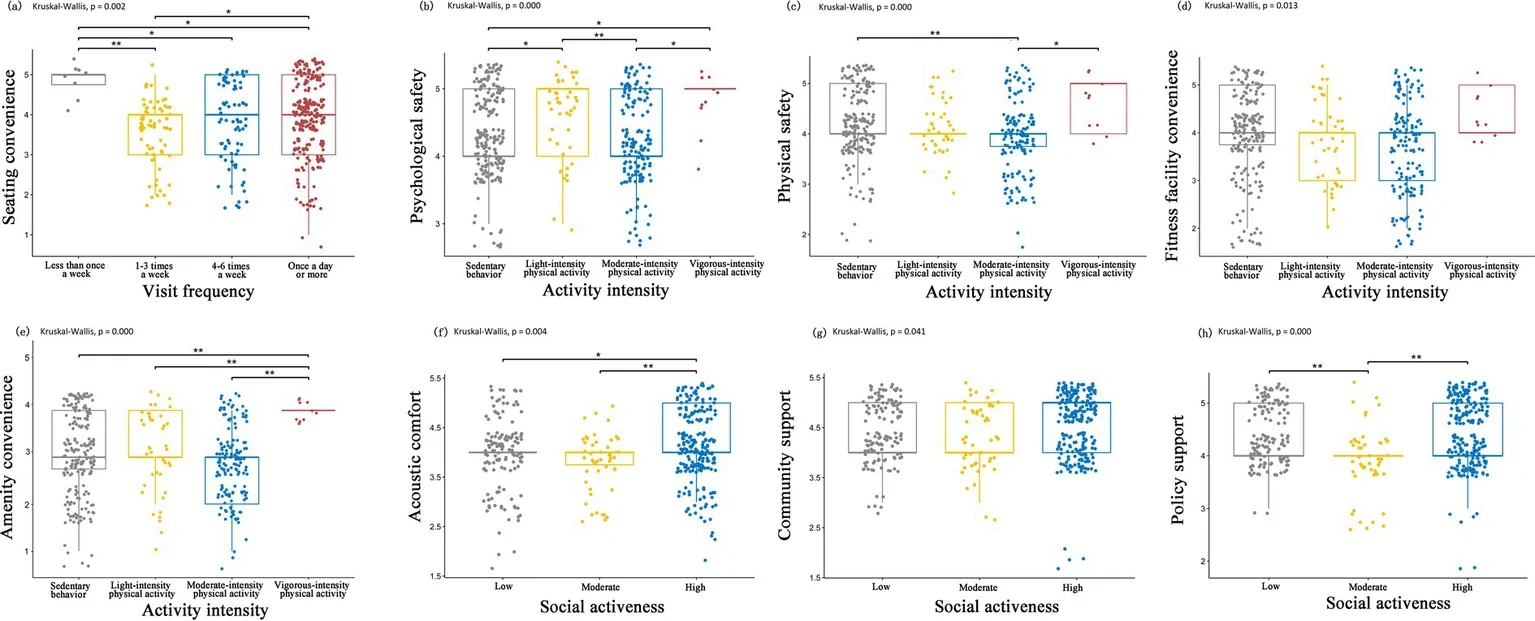
Post-hoc multiple comparisons across different park exposures (*p < 0.05, **p < 0.01). **(a)** Differences in seating convenience by visit frequency. **(b)** Differences in psychological safety by activity intensity. **(c)** Differences in physical safety by activity intensity. **(d)** Differences in fitness facility convenience by activity intensity. **(e)** Differences in amenity convenience by activity intensity. **(f)** Differences in acoustic comfort by social activeness. **(g)** Differences in community support by social activeness. **(h)** Differences in policy support by social activeness.

Older adults with different activity intensities exhibit differed in their perceptions of psychological safety, physical safety, and fitness facility convenience, and amenity convenience. The psychological safety of individuals who engage in sedentary behavior or moderate-intensity physical activity is significantly lower than that of those who engage in light- or vigorous-intensity physical activity (Figure 6b). The physical safety of individuals who engage in sedentary behavior or vigorous-intensity physical activity is significantly higher than that of those who engage in moderate physical activity (Figure 6c). Those who engage in moderate-intensity physical activity rated the fitness facility convenience lower than those who remained sedentary (Figure 6d). Vigorous-intensity physical activity was associated with significantly higher ratings of amenity convenience compared to other groups (Figure 6e).

Older adults with varying levels of social activeness differ in their perceptions of acoustic comfort, community support, and policy support. The group with high social activeness rated the acoustic comfort significantly higher than the other groups (Figure 6f), while the group with low social activeness rated policy support significantly lower than the other two groups (Figure 6h). Although there are overall differences in community support, the differences between groups are not significant (Figure 6g).

### 3.4 Linear regression analysis

The variance inflation factors for all four models are less than 5, indicating that there is no multicollinearity in the models. Table 9 presents the results of the multiple linear regression analyses for Model 1 (objective environment - composite physical activity index) and Model 3 (objective environment - composite social activeness index). The adjusted *R*^2^ values for Model 1 and Model 3 were 0.12 and 0.19, respectively, and the F-test was significant (*p* < 0.001), indicating that the models are generally valid.

**Table 9:** Multiple linear regression analysis of objective environmental factors and park exposure levels.

Variable	Model 1: objective environment-composite physical activity index			Model 3: objective environment-composite social activeness index			VIF
	*β*	*t*	*p*	*β*	*t*	*p*	
(Intercept)		3.498	<0.001^**^		4.570	<0.001^**^	
DI	−0.247	−3.652	<0.001^**^	−0.242	−3.714	<0.001^**^	1.047
NO	0.059	0.842	0.401	0.024	0.355	0.723	1.107
SL	−0.08	−1.178	0.240	−0.093	−1.421	0.157	1.054
AR	−0.079	−1.016	0.311	−0.151	−2.016	0.045^*^	1.396
GVI	−0.028	−0.402	0.688	−0.138	−2.065	0.040^*^	1.104
BVI	−0.037	−0.485	0.629	−0.131	−1.769	0.079	1.350
SVF	0.037	0.498	0.619	−0.044	−0.626	0.532	1.246
SF	0.032	0.404	0.687	0.189	2.463	0.015^*^	1.453
AM	−0.025	−0.339	0.735	−0.147	−2.035	0.043^*^	1.282
RF	0.178	2.574	0.011^*^	0.086	1.301	0.195	1.092
AC	0.132	1.809	0.072	0.029	0.411	0.682	1.219
Gender	0.161	2.326	0.021^*^	0.204	3.075	0.002^**^	1.092
Age	−0.004	−0.059	0.953	−0.084	−1.245	0.215	1.123
Education level	0.102	1.476	0.142	0.056	0.841	0.402	1.080
Adjust *R*^2^	0.12			0.19			
Sig.	<0.001			<0.001			

*p < 0.05; **p < 0.01.

In Model 1, travel distance (*β* = −0.247, *p* < 0.001) was significantly negatively associated with composite physical activity index. The coefficient for fitness facility density was 0.178 (*p* < 0.05), meaning that, after controlling for other variables, a 1-unit increase in fitness facility density was associated with a 0.178-unit increase in composite physical activity index. None of the other variables were significant. In Model 3, seating density (*β* = 0.189, *p* < 0.05) was the only independent variable that had a significant positive effect on composite social activeness index. Meanwhile, travel distance (*β* = −0.242, *p* < 0.001), fitness facility density (*β* = −0.147, *p* < 0.05), size of sub-area (*β* = −0.151, *p* < 0.05), and green view index (*β* = −0.138, *p* < 0.05) all had a significant negative impact on composite social activeness. The effects of gender on the composite physical activity index and composite social activeness index were statistically significant, with females exhibiting higher exposure levels than males.

The results of the model indicate that perceived environmental factors have only a very limited predictive power regarding park exposure levels. Model 2 (perceived environment -composite physical activity index) was not statistically significant (*p* = 0.197, adjusted *R*^2^ = 0.019), and apart from the control variable gender (*β* = 0.203, *p* < 0.01), the regression coefficients for all independent variables were not significant (*p* > 0.05). This suggests that, within the sample and measurement framework of this study, perceived environmental factors do not effectively predict older adults’ park exposure levels.

## 4 Discussion

It is important to understand how older adults perceive the physical environment, as this can lead to greater satisfaction with community parks through more manageable and subtle interventions. Previous studies have mostly confirmed that better perception of the environment among older adults may promote park use and contribute to improved restoration, well-being, and overall health (Lo and Jim, 2010; Peschardt and Stigsdotter, 2013). However, this study found a systematic disconnect between older adults’ perception of the park environment and their actual use behavior. This is manifested at three levels: (1) environmental perception does not fully correspond to objective environmental factors; (2) environmental perception varies with personal characteristics and use contexts; and (3) perception has no significant predictive effect on exposure levels. Within the sample and variable scope of this study, this finding challenges the linear assumption that “improving the environment - enhancing perception - promoting use”, suggesting that research on park exposure among older adults needs to further consider the role of behavioral decision-making mechanisms (such as habits and cognitive dissonance), and that the boundaries of its applicability should be tested in broader samples and contexts in the future.

At the first level, older adults’ perception of the park environment is mainly manifested in two forms: relatively accurate cognitive mapping of certain factors, and biased cognitive transformation of other factors. The perceived convenience of seats and amenities among older adults was positively correlated with the actual numbers, reflecting that they had a relatively realistic and accurate cognitive mapping of the objective environment. Cognitive translation refers to a situation where older adults’ perception of a factor has no significant relationship with the objective condition of that factor itself but is associated with other objective environmental factors. This is mainly manifested in the close association of multiple perceptual factors with blue view index (BVI) and seating density; that is, being close to water and being able to view water features more clearly help improve older adults’ overall perceptual experience. Previous studies have also found that older adults favor waterscapes regardless of water feature type (Samadi-Todar and Hami, 2025; Van Puyvelde et al., 2025). The BVI can also strengthen their perception of soundscape comfort (Tian et al., 2023). In addition, various studies have highlighted the positive impact of the number and comfort of seating facilities on older adults’ park activities (Veitch et al., 2020). Regardless of whether older adults engage primarily in sedentary or active physical activities in the park, their declining physical function and mobility make them more reliant on seating facilities (Lee and Ho, 2021; Tao et al., 2025b). Therefore, BVI and seating density, as positively correlated factors, can lead older adults to form an “overestimated” perception of the objective environment, making them effective factors for improving overall satisfaction.

Conversely, SVF and travel distance may diminish or offset the positive benefits that other factors provide for older adults. In the hot weather of subtropical cities, shade is considered to be the most desirable feature of outdoor spaces by older adults (Lonn et al., 2025). SVF is the only objective factor that shows a significant negative correlation with other factors, indicating that a lack of shade significantly reduces older adults’ perception of the usability of facilities. In addition, the longer the travel distance, the lower the perceived level of community support. This is because the higher the time, physical, and transportation costs individuals face when traveling to the park, the more likely people are to subjectively feel that the “support” is difficult to obtain or irrelevant to themselves (Fang et al., 2025), thereby lowering their evaluation of community support.

At the second level, perceived environment is not only based on objective conditions but is also influenced by individual characteristics and park exposure status. This is consistent with the results of other studies (Ball, 2006; Huang and Lin, 2023). Compared to gender and age, differences in educational levels exhibit greater variation in perception among older adults. Higher education levels were associated with lower perceptual evaluations of the acoustic environment, fitness facilities, amenities, and support services, indicating that these groups have higher expectations for the functional support and community cohesion atmosphere of parks. Previous studies support this finding, as more highly educated individuals participate more frequently in physical activities and hold higher expectations for park facilities (Mobily, 2014; Machac et al., 2022). This study also found that women reported a higher sense of safety than men. This differs from conventional perceptions (Samadi-Todar and Hami, 2026). This may be because previous studies were mostly conducted in communities with higher crime rates, whereas Chinese cities have a relatively high level of public safety, and park users are more concerned about risks arising from the environment or their own physical conditions rather than external threats. Baran et al. (2018) also found no significant gender differences in the sense of safety in open environments. In this study, women’s higher sense of safety may be related to their stronger social connections—women accounted for 60.2% of the socially active group. As for older adults being the group with a higher sense of psychological safety, this can be attributed, on the one hand, to the “paradox of aging”, whereby the oldest older adults paradoxically exhibit higher life satisfaction and psychological resilience as they age (Lee, 2025). On the other hand, those older adults who are able to continue visiting parks tend to have greater awareness of the park environment and a stronger sense of place attachment (Jang et al., 2024), thereby forming higher perceptual evaluations (Shabani et al., 2026).

With regard to usage patterns, some studies have found that as park features are perceived more frequently, their role in enhancing satisfaction diminishes until it disappears (Yang and Wu, 2025). However, this study identified nonlinear patterns that are inconsistent with the above findings. Seating convenience varies significantly with visit frequency, exhibiting a “U-shaped” pattern. This may be because newcomers focus more on the overall experience and the novelty of it, making them more likely to form a peak experience. This has also been confirmed in previous studies (Zhan et al., 2025). Meanwhile, frequent visitors tend to have relatively consistent patterns of use over time and space; their long-term, continuous use of the sub-area and seating has fostered a strong sense of territory and control, giving them an advantage in resource utilization. Visitors who fall between these two groups are familiar with the sub-area, but their use of the facilities is significantly less intensive than that of daily visitors. Their perceptions are driven more by experience and practicality; while they can identify shortcomings in the objective environment with greater accuracy, they are also likely to develop “sensory adaptation” and esthetic fatigue (Yang and Wu, 2025; Zhan et al., 2025). Apart from seating, no similar differences were observed in the perception of other factors, suggesting that suitable seating is a resource of widespread concern among older adults in the park. Differences in older adults’ perception of the environment are also closely related to their use behavior. Specifically, they tend to regard factors related to their activity preference as “focal point” and thus give them lower ratings, while possibly overestimating factors unrelated to their activity preference and treating them as “background.” For example, moderate-intensity physical activity relies more on fitness facilities, so this group’s evaluations of such facilities are significantly lower than those of the sedentary group. This indicates that perceptual evaluation is not a direct mapping of the objective environment, but rather a constructed evaluation filtered through “preference relevance.” The above perception formation mechanism not only explains why perceptual evaluations deviate from the objective environment, but also provides a premise for understanding the disconnect between perception and behavior. That is, when perceptual evaluation itself has already been reconstructed by use behavior, it can hardly serve as an external predictor of behavior anymore.

At the third level, in this study, perceived environmental factors did not show a significant predictive effect on older adults’ level of park use. Linear regression analysis indicated that there was no significant linear relationship between perceptual evaluation and frequency of use. This result suggests that there is a systematic disconnect between perception and behavior. As other studies have pointed out, the disconnect between perceived environment and behavior is more pronounced among older adults than among younger people. It manifests in two ways: first, higher satisfaction does not necessarily mean higher frequency of use; second, low satisfaction can hardly prevent habitual use (Rui and Gong, 2026); that is, older adults may maintain stable use behavior despite low evaluations of the environment. At the theoretical level, this disconnect can be explained by habitual behavior theory and cognitive dissonance theory. On the one hand, when older adults who have lived in a place for a long time visit a park repeatedly every day, their exposure behavior relies more on habit and is no longer driven by attitudes or perception (Rui and Gong, 2026). Place attachment and familiarity may lead them to subjectively downplay environmental deficiencies and maintain high-frequency visits (Hui et al., 2026). On the other hand, the mechanism of reducing cognitive dissonance enables older adults, when unable to change the objective environment, to reduce psychological discomfort by actively lowering their expectations (Carp, 1975), thereby maintaining satisfaction with the environment, which in turn is externalized as more tolerant evaluations of the environment. Therefore, perceptual evaluation may reflect “adaptive satisfaction” rather than a genuine evaluation of the environment. Nevertheless, the results of this study do not deny the importance of environmental perception, but rather suggest that its role may be more indirect and complex. Although the perceptual factors included in the study showed inconsistent patterns of covariation with older adults’ behavior, perceived environment may still affect psychological experience and, as a contextual factor influencing behavior, exert its effects through interactions with personal motivation, habits, and other factors.

The findings of this study support the argument that the objective environment plays a more significant role in park exposure (Tao et al., 2025a). However, the model’s overall explanatory power is quite limited, largely because park exposure is influenced by complex, multifactorial factors; other studies have also found that park features have a relatively minor impact (Li et al., 2021; Wang et al., 2022). However, we still identified several significant factors from the model that have direct implications for planning practice. Travel distance has a significant negative impact on exposure levels in both dimensions; longer travel distances reduce accessibility and likely hinder physical activity and social interaction by decreasing visit frequency (Fry et al., 2024; Tao et al., 2025a). For urban planning, this means that park green spaces should be incorporated into the “15-minute living circle” system, especially in high-density aging communities, where small and micro park spaces should be prioritized to fill service blind spots. A higher density of fitness facilities promotes physical activity, while a higher density of seating promotes social interaction, confirming that the park exposure level depends on the provision of closely related facilities. Therefore, facility provision should match the target behavior: areas oriented toward physical activity should ensure the accessibility of fitness equipment, while areas oriented toward social interaction should provide sufficient seating. In summary, this study shows that park exposure level depends not only on the quantity of facilities but also on the way facilities are configured and spatially organized to match older adults’ activity needs. Therefore, age-friendly park development should shift from “quantity provision” to “demand matching,” incorporating older adults’ activity characteristics as a core basis for planning and design.

This study has the following limitations: (1) The non-significant prediction of perceived environment on older adults’ park exposure level revealed in this study has its explanatory power strictly limited by the sample characteristics and variable operationalization definitions of this study. This does not deny the influence of perceived environment in other populations or contexts, but merely indicates that within the current research framework, objective structural factors (such as travel distance and facility density) may play a more prioritized screening role in older adults’ behavior. Therefore, we advocate treating this conclusion as a context-dependent finding, whose external validity remains to be verified and extended in larger-scale, multi-temporal studies. (2) The study also has limitations in sample selection. For reasons of survey ability, data collection focused on community park sub-areas where older adults are relatively active, and did not include sub-areas with lower frequency of use. Although this ensured that the study focused on park environments with actual use functions, it also means that the conclusions are not suitable for direct generalization to park sub-areas that remain underused or abandoned for long periods. (3) We fully recognize the cross-sectional nature of this study in the temporal dimension. Therefore, the primary contribution of this study’s conclusions lies in revealing the patterns of park exposure under the specific social-ecological context of “summer daytime,” rather than attempting to establish universal laws across seasons and time periods. Although the findings of this study may not be directly extrapolated to other seasons or nighttime contexts, they precisely provide testable benchmark hypotheses and comparative baselines for subsequent research on spring, autumn, and winter as well as day-night comparisons. We recommend that future studies adopt multi-temporal repeated measures or ecological momentary assessment methods to systematically disentangle the interactive moderating effects of season and time period on exposure perception. Since the data were collected during summer daytime, the conclusions are mainly applicable to explaining the patterns of park exposure perceived during summer daytime. These findings cannot be generalized to nighttime activities or other seasons. (4) Although the model incorporating objective environmental factors is statistically significant, its overall explanatory power for park exposure level is limited, and its predictive performance is not ideal. This indicates that the independent variables selected in this study are insufficient. Future research could further collect data from other seasons and nighttime periods, and, on the basis of expanding the sample and variable scope, conduct nonlinear analyses to build a more systematic research framework, thereby more comprehensively revealing the influencing factors of park exposure level and their possible threshold effects.

## 5 Conclusion

This study was conducted in community parks in Xiamen, China. It examined the perceptual characteristics of older adults regarding the objective park environment and compared the effects of the objective environment and environmental perception on park exposure. The findings show that older adults’ perceptions of seating and service facilities were relatively consistent with actual conditions, whereas their perceptions of acoustic comfort, safety, fitness facilities, and community support were mostly significantly positively correlated with the blue view index and seating density, and significantly negatively correlated with SVF and travel distance. This indicates that the BVI and seating density are positive factors that enhance overall satisfaction, while SVF and travel distance are negative factors that reduce overall satisfaction and may weaken the perceptual effects of other factors. Second, perceptual evaluations exhibited significant group differences: older adults with higher education levels often showed lower perceptual evaluations, whereas women and the oldest group (80–90 years) rated safety perception more highly. Different visit frequencies and activity intensities also showed significant perceptual differences for some factors. In addition, older adults tend to regard environmental factors related to their preferred behaviors as “focal points” and thus give them lower ratings, while they may overestimate environmental factors unrelated to their preferred behaviors. Notably, environmental perception in this study had no significant predictive effect on older adults’ park exposure levels, suggesting a systematic disconnect between perception and behavior—that is, low evaluations are unlikely to prevent habitual use, and high satisfaction does not necessarily attract high-frequency use. This disconnect does not negate the role of perception in behavior; rather, it indicates that the behavior and attitudes of older adults may be moderated by factors such as habit and cognitive dissonance. Therefore, park intervention strategies derived solely from satisfaction surveys may not be directly effective in promoting older adults’ park exposure levels; they should be comprehensively evaluated by integrating objective environmental conditions, environmental perception, and older adults’ activity preferences through multidimensional data. This provides a new perspective for the planning and assessment of age-friendly parks that goes beyond a single satisfaction indicator.

## Data Availability

The original contributions presented in the study are included in the article/supplementary material, further inquiries can be directed to the corresponding author/s.
